# 

**Published:** 1897-01

**Authors:** 


### CONTENTS.

COMMUNICATIONS.

The Orbicularis Oris, and the Muscles of Expression........................ 1

PROCEEDINGS.

The Fate of Associations........................................................ 10
Discolored Structure of Devitalized Teeth—Causes, Method of Treatment and Agents Employed........................................ 20

MONTHLY DIGEST.

Cataphoresis—Early History........................................................ 32

SELECTIONS.

Decision on the Low Bridge Patent.............................................. 43

EDITORIAL.

Ohio State Dental Society........................................................ 47
Toledo Dental Society........................................................ 48
Southern Indiana Dental Society........................................................ 50
The Chicago Dental Society........................................................ 50
Enamel Polish........................................................ 51
Hygienic Resort........................................................ 51
Bibliographical........................................................ 52

